# Neo-RAS Wild Type or RAS Conversion in Metastatic Colorectal Cancer: A Comprehensive Narrative Review

**DOI:** 10.3390/cancers16233923

**Published:** 2024-11-22

**Authors:** Guido Pesola, Samantha Epistolio, Marco Cefalì, Elena Trevisi, Sara De Dosso, Milo Frattini

**Affiliations:** 1 Oncology Institute of Southern Switzerland (IOSI), Ente Ospedaliero Cantonale, 6500 Bellinzona, Switzerland; guido.pesola@eoc.ch (G.P.); marco.cefali@eoc.ch (M.C.); elena.trevisi@eoc.ch (E.T.); sara.dedosso@eoc.ch (S.D.D.); 2 Laboratory of Genetics and Molecular Pathology, Istituto Cantonale di Patologia EOC, 6600 Locarno, Switzerland; samantha.epistolio@eoc.ch; 3 Faculty of Biomedical Sciences, Università della Svizzera Italiana, 6900 Lugano, Switzerland

**Keywords:** RAS conversion, neo-RAS wild type, liquid biopsy, colorectal cancer, bevacizumab

## Abstract

The management of metastatic colorectal cancer in patients harboring RAS mutations primarily involves chemotherapy, often combined with bevacizumab, as a standard first-line treatment. However, emerging evidence suggests that tumors in a subset of these patients may experience a conversion from RAS-mutant status to RAS wild type (wt) during or after chemotherapy, a process referred to as “RAS conversion” or “neo-RAS wt”. Understanding the mechanisms driving the neo-RAS wt phenomenon is crucial for its application in personalized medicine. Hypotheses suggest that selective pressure from chemotherapy may lead to a decrease in the number of mutant RAS clones or an outgrowth of pre-existing RAS wt clones. Further research is needed to validate these mechanisms and understand the impact of the neo-RAS wt phenomenon on long-term outcomes, such as overall survival and progression-free survival. This review provides a comprehensive overview of the current understanding of the neo-RAS wt phenomenon, including its incidence, potential mechanisms, and clinical implications.

## Simple Summary

Patients with RAS-mutant metastatic colorectal cancer are typically treated with chemotherapy, with or without bevacizumab, as the first-line therapy. Over time, tumors in some patients may undergo plasma clearance of RAS, transitioning from being RAS-mutant to RAS-wild type, a phenomenon known as “RAS conversion” or “neo-RAS wild type”. The current review focuses on this phenomenon’s incidence, evaluation methodologies, and therapeutic implications, with a focus on the role that bevacizumab plays in it and its prospects.

## 1. Introduction

Mutations in RAS genes are common in patients with colorectal cancer (CRC), occurring in nearly 40% of all CRC cases, and result in resistance to treatment with epidermal growth factor receptor (EGFR) monoclonal antibodies [1,2]. Therefore, investigating the mutational status of the RAS gene is crucial for selecting the optimal treatment combination for CRC [3]. Standard first-line systemic therapy for RAS-mutant metastatic CRC (mCRC) typically involves fluoropyrimidine-based chemotherapy with the addition of the anti-angiogenic drug bevacizumab. This drug has been shown to increase overall survival (OS) (hazard ratio [HR]: 0.79; 95% confidence interval [CI]: 0.69–0.90; *p* = 0.0005), progression-free survival (PFS) (HR: 0.63; 95% CI: 0.49–0.81; *p* = 0.0004), and the response rate (RR: 1.50; 95% CI: 1.06–2.10; *p* = 0.02) in this population [4].

Even though most RAS-mutant patients treated with systemic therapy tend to remain mutated over time [5], clonal evolution can occur in some cases, leading to the disappearance of RAS-mutant clones [6,7,8,9,10,11,12,13].

Current evidence on clonal evolution primarily relies on liquid biopsy assessment, particularly plasma samples from which circulating tumor DNA (ctDNA) has been extracted. Exceptions include the studies by Epistolio et al. and Arici et al., where mutations were evaluated in resected metastases [14,15].

The liquid biopsy approach offers significant advantages: being minimally invasive, it can be repeated serially over time, and it allows real-time monitoring of tumor recurrence, metastasis, or therapeutic response, as has been widely demonstrated in recent years [6,7,8,9,11,12,16,17,18].

Various molecular liquid biopsy-based techniques have been considered for the treatment of CRC, such as the evaluation of circulating tumor cells, ctDNA, exosomes, and tumor-educated platelets [17]. These techniques are showing promising results in ongoing trials for choosing therapeutic strategies and predicting the recurrence or prognosis of CRC [17,19].

The disappearance of RAS clones in the plasma of patients diagnosed with RAS mutations is known as “neo-RAS wild type (wt)” or “RAS conversion”. Current scientific evidence regarding this phenomenon is mixed. The concept of “neo-RAS wt” has not been unambiguously defined, partly due to the different thresholds and cut-off points applied to the methods and technologies that have been employed for liquid biopsy analysis used so far (e.g., quantitative PCR [qPCR, TaqMan methodology], Ion Torrent^TM^ [Thermo Fisher Scientific, Waltham, MA, USA], Idylla^TM^ [Biocartis, Melchen, Belgium], MassARRAY System^®^ [Agena Bioscience, San Diego, CA, USA], Guardant360^TM^ [Guardant Health, Palo Alto, CA], OncoBEAM^TM^ [Sysmex Suisse, Horgen, Switzerland], and HapOnco CDx^TM^ [Haplox Biotechnology, Hong Kong]). Each different methodology is based on its own limits of detection (LODs) for the RAS mutation, making it difficult to provide a global definition of the neo-RAS wt phenomenon.

In this narrative review, we aim to summarize the current literature regarding the neo-RAS wt phenomenon. Specifically, our objective is to cover this phenomenon’s incidence rates, evaluation methodologies, and therapeutic implications, with a focus on the impact of bevacizumab on it and its prospects in this field.

## 2. General Considerations and the Role of RAS Mutations in CRC

CRC is the third most common cancer worldwide, accounting for approximately 10% of all cancer cases, and the second leading cause of cancer-related deaths. In 2022, global cancer statistics reported 1,926,118 new CRC cases [20]. When CRC is diagnosed at an advanced metastatic stage, treatment options are limited, and the five-year OS rate is lower than 20% [21]. Despite many cancer treatment advances, the therapeutic approach for stage IV CRC is generally not curative, focusing instead on increasing OS with an acceptable quality of life [3].

The introduction of specific anti-EGFR therapies, in the form of monoclonal antibodies, represented an improvement in the treatment of advanced stages of CRC. However, it soon became clear that molecular alterations in EGFR alone are insufficient to distinguish which patients would benefit from these therapies and which would not, mainly due to the molecular complexity of CRCs [22].

Analyses of not only EGFR but also all the downstream pathways have revealed how underlying markers can signify resistance mechanisms to anti-EGFR therapies.

Some of the most relevant mechanisms of resistance to anti-EGFR drugs are mutations in the RAS family genes. These mutations activate the RAS proteins and, consequently, continuously simulate downstream pathways, resulting in cell proliferation and survival. This constitutive activation can suppress the efficacy of anti-EGFR therapies [23]. KRAS mutations are present in nearly 40% of CRC cases, and the prognostic role of this gene is closely associated with the localization of these mutations and the specific mutation variants [23]. It has been demonstrated that RAS mutations are associated with a worse prognosis compared to wt (25.8 months versus 35.1 months, respectively; *p* = 0.006), especially those that occur in codon 12 when compared to codon 13 (22.4 months versus 24.8 months, respectively) [24]. The main studies that investigated the interplay between EGFR downstream alterations and the efficacy of EGFR-targeted therapies were the CRYSTAL, OPUS, and PRIME trials [25]. The first two clinical trials led to the approval of the EGFR antibody cetuximab for the treatment of mCRC patients, showing that the combination of cetuximab with chemotherapy (FOLFIRI or FOLFOX) in patients whose tumors were KRAS wt improved OS (HR: 0.81; *p* = 0.0062), PFS (HR: 0.66; *p* < 0.0001), and ORR (OR: 2.16; *p* < 0.0001) compared to chemotherapy alone [25,26].

Regarding the monoclonal antibody panitumumab, the PRIME phase III study showed that treating KRAS wt patients with this drug along with FOLFOX in the first-line treatment improved median PFS compared to FOLFOX alone (8.6 months [95% CI: 7.5–9.5 months] versus 10.0 months [95% CI: 9.3–11.4 months], respectively) [27]. Similar results were obtained in the second line with an improvement in PFS comparing panitumumab plus FOLFIRI versus FOLFIRI alone (5.9 versus 3.9 months; HR: 0.71; *p* = 0.004) [28].

This body of evidence led international agencies (FDA and EMA) to approve the administration of cetuximab and panitumumab in only KRAS wt cases.

Therefore, KRAS-mutant patients are usually excluded from treatment with EGFR-targeted therapies. However, the development of drugs specifically targeting KRAS p.G12C mutations has prompted consideration of combining anti-EGFR therapies with KRAS-specific inhibitors in patients with the KRAS p.G12C mutation. Preliminary results from clinical trials have shown that direct inhibition of KRAS p.G12C is now possible, potentially leading to the development of a novel targeted treatment for several patients with advanced CRC [29]. Indeed, the combination of a KRAS p.G12C inhibitor and an anti-EGFR treatment has been demonstrated to improve PFS versus standard care among patients with chemo-refractory mCRC harboring a KRAS p.G12C mutation in the Code Break 300 and KRYSTAL-1 trials [30,31].

Recent advances in personalized medicine for CRC have focused on refining the selection of patients who may benefit from targeted therapies. A key aspect of this approach involves hyper-selecting patients based on specific molecular alterations, as evidenced in the usage of PRESSING panels, which incorporate an expanded range of genetic markers to guide treatment decisions [32,33]. Additionally, ctDNA analysis has emerged as a valuable tool in clinical trials, such as the PARADIGM and FIRE-4 studies, where it has demonstrated the ability to further refine patient selection, specifically by identifying patients unlikely to respond to anti-EGFR therapy [34,35]. These strategies represent a significant step forward in tailoring treatments to individual patients, potentially improving outcomes in CRC therapy.

## 3. Neo-RAS wt: A New Reality

CRCs are highly heterogeneous tumors characterized by cells harboring different mutational profiles [36].

However, molecular concordance between biopsies of the primary tumor and the tumor during metastasis is very high at the time of diagnosis, often exceeding 90% of cases, as shown in a meta-analysis [37].

The presence of heterogeneity necessitates a methodology capable of fully assessing the different types of genetic alterations present in these tumors. It is well known that tumors can release DNA directly into the circulatory stream as ctDNA or via circulating tumor cells or exosomes. The introduction of ctDNA analysis has allowed for a comprehensive overview of cancer genetics as well as clonal evolution of CRC [34,38]. It is worth noting that 20% of CRCs do not secrete ctDNA, particularly in patients with lung metastases or peritoneal carcinosis [39,40]. Consequently, this subgroup could present non-representative results from liquid biopsy analyses. A potential advantage of liquid biopsy is its ability to provide a more comprehensive snapshot of tumor dynamics and genetic alterations over time, offering insights that a single tissue biopsy sample may not capture. This is because liquid biopsy does not suffer from the sampling bias that can affect tissue biopsy, which may capture only a subset of the tumor’s heterogeneous cell population.

A concept that has gained significant relevance in recent times is the disappearance of RAS mutations observed in ctDNA from plasma over the course of the disease. This phenomenon has been referred to by various names, such as RAS reversion or RAS conversion, but it is now more commonly known as “neo-RAS wt”. A biological explanation for this phenomenon has yet to be defined in the literature. Many attempts have been made to find a biological explanation for the concerns associated with the neo-RAS wt transformation, but none of them has fully explained all the aspects of this phenomenon until now.

The possibility that anti-cancer therapy can exert pressure on mutant RAS cell clones is supported by two publications on tissue analysis: the first from our group, which showed a reduction in the variant allele frequency of RAS mutations in resected liver metastases from bevacizumab-based systemic therapy-pretreated patients [15], and the second by Arici et al., which showed that 9.3% of their resected metastases had lost the RAS mutation [14]. These results suggest that it could be very useful for patients to assess the RAS mutational status after first-line treatment and that liquid biopsy represents the less invasive technique as compared to tissue biopsy, although the analysis of a tissue biopsy can also be performed if there is a need for a histological evaluation of the disease for proper clinical management.

The change in the RAS mutational status may be explained by the hypothesis that systemic therapy administered to patients affected by KRAS-mutant mCRC can have a better effect on KRAS-mutant cells even if this treatment does not include specific KRAS inhibitors. Consequently, a tumor majorly characterized by KRAS-mutant clones at diagnosis may become mostly KRAS wt. The motivation behind this selection is, to date, a matter of debate and has not been clearly described in the literature. Many biological pathways may be influenced by systemic therapy that can cause clonal selection of some molecular features, but no definitive demonstration of this has been reported so far in the literature.

The disappearance of an RAS mutation in the absence of systemic treatment could account for the percentage of patients for whom there is no concordance between primary tumor and metastasis. This discrepancy of plasma versus tissue might also be imputable to the limitations of ctDNA detection or spiral and temporal heterogeneity in RAS-mt tumor clones within the tumor issue or other aspects such as long intervals between assessments of the molecular status in tumor tissue and ctDNA, resection of the primary tumor at the time of blood draw, tumor site, and type of tissue analyzed [39].

Nonetheless, it should be noted that in the current evidence on the neo-RAS wt phenomenon, the presence of circulating mutations prior to the initiation of systemic therapy was not systematically reported. Reports from clinical trials are less affected by this bias [11,41,42,43,44,45].

In studies published between 2019 and 2024, the incidence of patients presenting with RAS regression is highly variable [5,6,11,13,18,21,41,42,43,44,45,46,47]. All authors who have explored the incidence of this phenomenon with different methodologies have highlighted this, albeit with very variable frequencies, ranging from 5.5 to 78%.

We have summarized the studies that reported having at least 50 patients in Table 1.

One of the most scientifically productive groups to have described this phenomenon is that of Nicolazzo and colleagues, who produced a total of four reports between 2019 and 2023 [9,18,49,50]. In their latest report in 2023, they described the presence of neo-RAS wt in 60% of the cases they analyzed, with 42 out of 70 patients given the first-line treatment followed longitudinally with Idylla (Biocartis). A high incidence was also reported by Wang and colleagues using HapOncoCDx (Haplox Biotechnology); they reported that 42.6% of 61 patients were given first-line treatment [43]. On the other hand, Osumi and colleagues reported the largest population based on the nationwide Japanese screening platform SCRUM-Japan GOZILA regarding patients with an initial diagnosis of RAS-mutant mCRC in different lines of therapies; the cfDNA test Guardant 360 highlighted a prevalence of 19.0% (91/478) of neo-RAS wt in Group A (all eligible patients) and 9.8% (42/429) in Group B, a subgroup with at least one somatic alteration detected in plasma [48]. Henry and colleagues at the MD Anderson Cancer Center found a low incidence of neo-RAS wt, between 2 and 8%, in two cohorts, analyzing a total of 236 patients in all line therapy settings [41].

Many confounding factors and biases can explain the large differences in terms of incidence, apart from the ctDNA methodology aspect, which will be discussed in the next section.

One critical aspect concerns the study population of the studies shown in the table, which is very heterogeneous.

Patients treated in the first-line setting have a higher incidence, as reported in the JACCRO CC-11 trial by Sunakawa et al., the clinical trial of Wang et al., and the series reported by Nicolazzo et al. [18,43,44]. However, the PLACOL study reported by Moati et al., which included patients in first-line treatment, reported low conversion rates [42].

In contrast, the reports by Henry, Osumi, and Wu covered more heterogeneous case histories with patients in various lines of treatment. In this case, the neo-RAS-wt rates were lower [16,41,45].

From an overall non-systematic evaluation of the studies, patients during the first line of therapy might have a greater chance of presenting a neo-RAS wt status. A possible biological rationale could be the smaller difference in terms of clonal heterogeneity and tumor burden in the first-line setting compared to heavily pre-treated patients.

On the other hand, inclusion in a clinical trial setting and the number of patients being high do not seem to be determining factors.

Furthermore, Osumi and colleagues described the characteristics of neo-RAS-wt patients with a regression multivariate analysis. They showed a higher conversion rate for patients without liver metastasis, smaller tumor diameter, and tissue RAS mutation other than KRAS exon 2 [16].

## 4. Methodologies for Neo-RAS wt Evaluation

Besides patient characteristics, treatment types, and the number of lines of therapies, another factor that can explain most of the differences observed in the neo-RAS wt status evaluation among the various studies published in the literature is the type of molecular tests applied to a given cohort. The reasoning behind this is that we are discussing the possibility of identifying mutations in liquid biopsies, and, to date, the only consensus among researchers regarding this is the use of a technique with “sufficient” sensitivity. Currently, there is no gold-standard method for analyzing ctDNA in plasma, nor is there a consensus on the minimum sensitivity required for the methodology. The methodologies available on the market are characterized by a wide range of sensitivities for identifying mutant alleles (not only KRAS), especially in contexts of high dilution with normal alleles and significant ctDNA degradation. The methodologies employed for investigating the presence of ctDNA are heterogeneous across all these studies, but all authors employed either qPCR (e.g., TaqMan PCR assays and Idylla^TM^, Biocartis), NGS (e.g., Ion Torrent^TM^, ThermoFisher Scientific; Guardant 360^TM^ and Guardant OMNI^TM^, Guardant Health; and HapOncoCDx^TM^, Haplox Biotechnology), ddPCR (e.g., OncoBEAM^TM^, Sysmex), or the MassARRAY^®^ System methodology (Agena Bioscience), which combines mass spectrometry with end-point PCR [5,6,9,11,16,18,21,41,42,43,44,45,46,47,49,51]. None of these methodologies is better or more sensible than the others because they are really different from each other regarding their strengths and limitations. The most common methodologies employ the Idylla^TM^ (Biocartis) and Guardant^TM^ technologies, both applied in three publications analyzing a total of six different populations, with the number of cases ranging from 23 to 82 for the IdyllaTM (Biocartis) and 95 to 478 for the Guardant^TM^ technology [13,18,21,41,45,46,52]. The Idylla^TM^ technology (Biocartis) has the strengths of having a high sensitivity (it can find nearly the totality of mutations analyzed—more than 73.9%) [5] and being easy to perform; however, this methodology can analyze only one marker for each experiment. A lower sensitivity (5.1–8.7%) [52] characterizes the Guardant^TM^ technology because, being an NGS methodology, this methodology analyzes different genes in a single run. The analysis of multiple markers decreases the sensitivity of the methodology, which is also an advantage of this methodology. A disadvantage of Guardant^TM^, when compared to Idylla^TM^ (Biocartis), is that it requires highly specialized personnel to carry out the experiment. Comparing the qPCR methods, we can affirm that Idylla^TM^ (Biocartis) has the same sensitivity as other TaqMan PCR methodologies but offers the advantages of being easier to apply and using less time from DNA extraction to mutation detection; it can complete this in a single step on a single cartridge. Another NGS methodology is Ion Torrent^TM^ (Thermo Fisher Scientific). Only two studies employed the NGS Ion Torrent^TM^ technique (Thermo Fisher Scientific) [42,47]. In one of these [42], even though the number of patients may be considered representative of a real-world population (n = 61), it detected a very low percentage of neo-RAS wt patients (3.3%). Like Guardant^TM^, the strength of Ion Torrent^TM^ (Thermo Fisher Scientific) is the analysis of multiple markers at one time, but this reduces the sensitivity of the methodology. Ion Torrent^TM^ (Thermo Fisher Scientific) has lower sensitivity compared to other techniques, such as MassARRAY^®^ (Agena Bioscience), OncoBEAM^TM^ (Sysmex), HapOncoCDx^TM^ (Haplox Biotechnology), the TaqMan methodology, and GuardantOMNI; studies employing these found, respectively, 56%, 20.9–83.3%, 15.2%, 51.6%, and 5.1–8.7% of neo-RAS wt patients [5,6,11,13,18,21,41,42,43,44,45,46,51]. In the paper by Moati et al., the Ion Torrent^TM^ (Thermo Fisher Scientific) methodology presents a neo-RAS wt patient rate (3.3%) closer to that of another NGS methodology described in the literature for neo-RAS wt determination: Guardant 360^TM^ or GuardantOMNI (5.1–8.7%) [13,41,42,45]. In the other study employing Ion Torrent^TM^ (Thermo Fisher Scientific), the neo-RAS wt rate was higher (36.4%), but no conclusions could be drawn about neo-RAS wt incidence due to the low number of cases analyzed (n = 11) [47]. In a small number of papers, the authors applied a second confirmatory method to assess the presence of ctDNA by methylation markers and evaluate the evolution of ctDNA release in the bloodstream [5,10,12,41].

Regarding the presence of evaluable ctDNA, the Idylla^TM^ (Biocartis), MassARRAY^®^ (Agena Bioscience), and Guardant360^TM^ methodologies (Guardant Health) found this marker in nearly all cases. In contrast, Ion Torrent^TM^ (Thermo Fisher Scientific), OncoBEAM^TM^ (Sysmex Suisse), HapOncoCDx^TM^ (Haplox Biotechnology), and qPCR detected the presence of ctDNA in 59%, 48%, 36%, and 66% of patients, respectively [5,6,11,13,18,21,41,42,43,44,45,46,47,51]. To sum up the advantages and disadvantages of all the methodologies reported above, we can conclude that all the NGS methodologies (e.g., Ion Torrent^TM^, ThermoFisher Scientific; Guardant 360^TM^ and Guardant OMNI^TM^, Guardant Health; and HapOncoCDx^TM^, Haplox Biotechnology) have the advantage of providing a broader evaluation of molecular markers than the other techniques, but for the same feature, they are characterized by lower sensitivity.

The KRAS mutational rates need to be considered based on the LOD of the specific methodology applied for the evaluation of RAS mutations. The LOD can be defined as the lowest concentration of the analyte that can be detected. In liquid biopsies, the LODs for the aforementioned assays are 2–5% for Idylla^TM^ (Biocartis), 2–10% for MassARRAY^®^ (Agena Bioscience), 2–10% for Guardant360^TM^ (Guardant Health), 1–2% for Ion Torrent^TM^ (Thermo Fisher Scientific), 1% for OncoBEAM^TM^ (Sysmex Suisse), 3–5% for HapOncoCDx^TM^ (Haplox Biotechnology), and 1–5% for qPCR [5,6,11,13,18,21,41,42,43,44,45,46,47,51]. As described here, the methodologies cited in the literature could have different LODs in liquid biopsies. The different LODs can influence the interpretation of the rates and presence of RAS mutations. To address this issue, future studies must confirm rather than exclude the presence of ctDNA in a plasma sample. Two approaches are possible: tumor-informed analyses or methodologies based on normalization. The first requires knowledge of the somatic mutations present in the primary tumor tissue and the investigation of the presence of somatic mutations in the ctDNA sample through next-generation sequencing (NGS) [42,47]. The second uses cancer-specific methylated biomarkers as a “normalizer” of the quantity of ctDNA available in plasma [10].

Considering these limitations, we can speculate that an accurate diagnostic method, combined with appropriate patient selection, could favor positive results, paving the way for the use of ctDNA even in patients with an initial RAS gene mutation. Analyzing the different diagnostic methods of the studies included in our review, we observe a high variety (MassARRAY [Agena Bioscience], Guardant360^TM^ [Guardant Health] or GuardantOMNI^TM^, OncoBEAM^TM^ [Sysmex Suisse], IonTorrent^TM^ [Thermo Fisher Scientific], Idylla^TM^ [Biocartis], and HapOncoCDx^TM^ [Haplox Biotechnology]) characterized by different sensitivities. Initially, we assumed that the different rates of neo-RAS wt ctDNA evaluable at follow-ups could be connected to the different methodologies applied and their varying sensitivities. However, by observing the individual methodologies, we found that the rate of neo-RAS wt cases detected is not comparable across the different studies (except for the Guardant360^TM^ (Guardant Health) or GuardantOMNI^TM^ assays). This suggests that it is mostly sample heterogeneity, rather than the use of a specific method, that defines the percentage of neo-RAS wt characterizable in CRC populations. Consequently, the proportion of reverted patients may not be homogeneous, with some cases being incorrectly defined as neo-RAS wt because the analysis involved only the subset of cells without mutations.

Considering sensitivity and feasibility, we can conclude that different methodologies need to be applied for specific clinical and practical scenarios. For example, if a patient is highly symptomatic and there is no possibility of obtaining the mutational landscape status in a short time, methodologies that are easier and faster to conduct are recommended for a first screening (e.g., Idylla^TM^, Biocartis). A more comprehensive analysis by an NGS methodology might instead be more recommended in the case of a patient with mutations that cannot be monitored by a smaller panel or in case the possible mechanisms of resistance to progression have to be investigated. In general, the most recommended methodology is qPCR by TaqMan technology [44]. Other methodologies with higher sensitivity (such as next-generation sequencing and ddPCR) have more complex protocols and require greater expenses for both instruments and reagents [5,11,51]. Some authors have addressed the problem of normalizing the real quantity of RAS mutations by employing a comparison with methylation profiles, as the application of circulating methylated DNA evaluation for defining the dynamics of RAS mutation clearance in plasma can be a reliable method for defining the real quantity of ctDNA in blood at different time points [5,10,12,41,42].

## 5. Does Bevacizumab Increase Neo-RAS wt Likelihood?

Another aspect we want to investigate further is whether specific drugs, such as anti-angiogenic compounds, may alter the neo-RAS wt rate. Although the addition of anti-angiogenic therapy has been shown to improve the effects of chemotherapy in terms of response and survival, it is unknown whether this drug can exert selective pressure on RAS-mutated clones [4].

Research conducted by our group demonstrated that bevacizumab has the biological potential to lead to clonal selection on the RAS mutation. In our retrospective analysis, patients who received bevacizumab had a greater reduction in the variant allelic frequency of RAS mutation in resected liver metastases when compared to operated patients who received standard chemotherapy but not bevacizumab (57.1% versus 8.3%) [15].

Supporting this hypothesis, Nicolazzo highlighted that 42 out of 56 patients treated with bevacizumab experienced a reversion of the RAS in ctDNA. Only two groups of authors assessed whether a statistical difference existed between the rates of neo-RAS wt cases considering the use of the drug. Klein-Scory et al. did not conclude that bevacizumab had an additive effect, while Nicolazzo et al. found that the inclusion of this anti-angiogenic drug is beneficial [5,18,49]. The discrepancies found in these works can be mainly associated with the different methodologies applied for KRAS investigation: ddPCR and OncoBEAM^TM^ (Sysmex) in the work by Klein-Scory et al. versus Ion Torrent^TM^ (Thermo Fisher Scientific) in the work published by Nicolazzo et al. Moreover, the different cohort sizes could have influenced the data obtained, leaning in favor of the results of the research by Nicolazzo’s group, which evaluated 72 patients, versus the study conducted by Klein-Scory et al., who examined 12 patients [5,49].

The biological explanation for the selective effect of bevacizumab on mutant RAS cells remains uncertain. Some hypotheses are focused on the role of bevacizumab in inflammation and neo-angiogenesis as preliminary findings in transgenic murine models [53,54,55]. Bevacizumab reducing angiogenesis could have a better effect against the clones most dependent on vascular contributions, which resulted in vitro, after stimulation of angiogenesis, as the KRAS-mutant ones. A molecular explanation was described in the work by Figueras et al., who proposed how KRAS mutations influencing the Raf-RAS-ERKs pathway activate the VEGF-A promoter, creating VEGF-A-associated vascularization [55].

Other hypotheses include the increased effect of bevacizumab-induced hypoxia on these clones or the drug’s ability to increase oxidative stress on mutant RAS cells [15,18].

These data suggest a potential role for bevacizumab as a drug that can increase the chance of achieving an RAS wt time window in which EGFR monoclonal antibodies can be administered. However, most current publications on neo-RAS wt do not allow for a comparison between patients treated with bevacizumab and those who did not receive the drug. We strongly recommend that researchers and clinicians considering the neo-RAS wt phenomenon in their clinical practice evaluate the potential role of bevacizumab in this context.

## 6. Neo-RAS wt: Possible Therapeutic Implications and Prospects

The evidence of neo-RAS wt has led clinicians to evaluate the use of anti-EGFR therapy in patients usually precluded from targeted therapy.

One of the first cases demonstrating the effectiveness of this therapeutic strategy was reported in the article by Gazzaniga et al. in 2018 [8].

To date, we can find in the literature some encouraging results regarding the use of EGFR inhibitors in neo-RAS wt patients. As summarized in Table 2, the authors of these studies presented responses and sustained PFS of EGFR inhibitor monotherapy alone or in combination with irinotecan for neo-RAS wt patients [7,10,11,12,16,47,48,52].

With the limitation of small sample sizes, keeping the number of patients reported by authors to a maximum of 10, the median PFS for anti-EGFR drugs ranged from 5.5 to 14.5 months. This is comparable to, or in some cases better than, the historical standard second-line treatment with anti-angiogenics such as FOLFIRI + aflibercept or FOLFIRI/FOLFOX + bevacizumab [56,57].

Even though anti-EGFR therapies have shown promising PFS in neo-RAS-wt patients, they have not been shown to increase OS when compared to standard regimens, which is a crucial endpoint in cancer treatment. Therefore, we do not consider these therapies to be usable outside of clinical trials.

These preliminary results are a good starting point for beginning to consider the efficacy of anti-EGFR therapies in neo-RAS wt cases. Some phase II trials combining chemotherapy and anti-EGFR are ongoing (Table 3). In most of them, the major confounding factors are the lack of a control arm. We see heterogeneity within these ongoing trials, particularly in the methods used to define mutations. Three trials will evaluate RAS status via OncoBEAM (Sysmex Suisse) and two via Idylla (Biocartis).

Looking ahead, we anticipate that future prospective clinical trials will not only confirm the effectiveness of this approach but also integrate comprehensive translational research involving both tissue samples and ctDNA.

Undoubtedly, an important aspect of future research will be understanding how to harmonize data from tissue analysis with ctDNA results to elucidate their clinical significance and better characterize tumor heterogeneity. The data obtained from the tissue will need to be harmonized more closely with those obtained from ctDNA analysis to give a clear picture of the mutational status of the RAS pathway during the course of the disease and treatment. This will be feasible by implementing ctDNA analysis, improving sensitivity, and comparing results from tissue analysis with the support of a molecular tumor board.

Discrepancies in results must be taken into account, and these should always be interpreted. A comprehensive analysis must take into account many aspects, such as patient characteristics, the type of medical treatment received and in progress, mutations highlighted by consideration of aspects such as neoplastic cellularity and allele frequency, and the limitations of the methods. Therefore, we believe that molecular tumor boards are essential for interpreting results and making clinical decisions.

## 7. Conclusions

At present, we are far from reaching definitive conclusions on the neo-RAS wt phenomenon, as the scientific community has yet to identify specific methods and patient profiles. The complexity of tumor biology and the heterogeneity of patient responses add additional layers of difficulty to this challenge.

Given these uncertainties, further studies are required to clarify the neo-RAS wt phenomenon. These studies should focus on not only expanding our understanding of the genetic and epigenetic factors involved but also refining diagnostic techniques to improve detection sensitivity. The development of more sensitive methodologies, such as enhanced sequencing technologies and comprehensive methylation profiling in ctDNA, holds promise for yielding more accurate and reliable results in the future.

Additionally, longitudinal studies and large-scale clinical trials are essential to identify potential biomarkers that can predict the emergence of neo-RAS wt and guide treatment decisions. Collaboration across research centers and the integration of multi-omics approaches will likely be crucial in overcoming the current limitations and moving towards more personalized and effective management of patients with CRC.

In conclusion, while significant progress has been made, the path forward requires a concerted effort to unravel the complexities of the neo-RAS wt phenomenon.

With ongoing research and the development of more sophisticated tools, we can anticipate a future in which the identification and management of this condition will become more precise, ultimately leading to improved patient outcomes.

## Figures and Tables

**Table 1:** Incidence of patients presenting with RAS regression and methodologies applied for identification of Neo-RAS wt from 2019 to 2024.

First Author, Year of Publication	Population (Real-World Prospective Study Versus Clinical Trial Versus Case Series)	Number of Patients	Type of ctDNA Analysis	RASmut Patients with Evaluable ctDNA Follow-Up	Neo-RAS wt Patients (n)	Neo-RAS wt Patients (%)
Henry, 2020 [41]	Real world	236	Guardant360^TM^ (Guardant Health)	202	12	6.0%
Moati, 2020 [42]	Real world	61	Ion Torrent^TM^ (Thermo Fisher Scientific)	36	2	5.5%
Wang, 2022 [43]	Real world	171	HapOncoCDx^TM^ (Haplox Biotechnology)	61	26	42.6%
Sato, 2022 [11]	Real world	129	OncoBEAM^TM^ (Sysmex)	62	27	43.5%
Sunakawa, 2022 [44]	Clinical trial	62	qPCR (TaqMan methodology)	41	32	78.0%
Nicolazzo, 2023 [18]	Case series	82	Idylla^TM^ (Biocartis)	70	42	60.0%
Osumi, 2024 [48]	Clinical trial	478 (group A) 429 (group B)	Guardant360^TM^ (Guardant Health)	478 (group A) 429 (group B)	91 (group A) 92 (group B)	19.8% (group A) 9.8% (group B)
Wu, 2023 [45]	Clinical trial	95	GuardantOMNI (Guardant Health)	95	6	6.3%

ctDNA: circulating tumour DNA; Neo-RAS wt: neo-RAS wild-type; RASmut: RAS mutated.

**Table 2:** Survival associated to administration of EGFR inhibitors in neo-RAS wt patients.

Author, Year	Patients (n)	Best Response	Survival (Months)
Bouchada, 2021 [7]	9	1 CR 4 PR 2 SD 2 PD	PFS: 8.2 OS: 22.3
Nicolazzo, 2021 [10]	10	NA	PFS: 10
Sato, 2022 [11]	4	1 PR 2 SD 1 PD	NA
Osumi, 2021 [12]	2	2 PR	NA
Osumi, 2023 [16]	6	1 PR 2 SD 3 PD	PFS: 4.8 (0.4–11.1)
Harada, 2023 [52]	2	2 PR	PFS: 5.5 (4–7)
Gramaça, 2024 [47]	4	NA	2nd line: PFS: 14.5 OS: 33.6 3rd line PFS: 3.9

NA: not available; CR: complete response; PR: partial response; SD: stable disease; PD: progressive disease; PFS: progression-free survival; OS: overall survival.

**Table 3:** Ongoing and closed phase II trials combining chemotherapy and anti-EGFR.

Study Name, Trial ID, Country	Method of ctDNA Analysis	Estimated Patients	Setting	Phase	Experimental Arm	Control Arm	Status
MoLiMoR, NCT04554836, Germany	OncoBEAM (Sysmex)	144	First line	II	FOLFIRI + Cetuximab	FOLFIRI + Bevacizumab	Active, not recruiting
CONVERTIX, EudraCT 2017-003242-25, Spain	OncoBEAM (Sysmex)	40	Second line	II	FOLFIRI + Panitumumab	NA	Closed
CETIDYL, NCT04189055, France	Idylla (Biocartis)	72	≥Third line	II	Cetuximab +/− Irinotecan	NA	Recruiting
KAIROS, EudraCT 2019-001328-36, Italy	Idylla (Biocartis)	112	Second line	II	Cetuximab + Chemo Doublet	NA	Recruiting
C-PROWESS, jRCT, s031210565, Japan	OncoBEAM (Sysmex) + Guardant360 (Guardant Health)	30	≥Second line	II	Panitumumab + irinotecan	NA	Recruiting

NA: not available.
